# Community Participation and Subjective Well-Being of Older Adults: The Roles of Sense of Community and Neuroticism

**DOI:** 10.3390/ijerph19063261

**Published:** 2022-03-10

**Authors:** Lanshuang Chen, Zhen Zhang

**Affiliations:** 1 Key Laboratory of Behavioral Sciences, Institute of Psychology, Chinese Academy of Sciences, Beijing 100101, China; chenls@psych.ac.cn; 2 Department of Psychology, University of Chinese Academy of Sciences, Beijing 100049, China

**Keywords:** community participation, subjective well-being, sense of community, neuroticism, older adults

## Abstract

Participation in community affairs and activities is beneficial to the mental health of older adults. The current study attempted to confirm the mediating role of sense of community (SoC) between community participation (CP) and subjective well-being (SWB), and the moderating role of neuroticism between CP and SoC. A total of 465 older adults aged ≥65 years from China participated in both two-wave online surveys. The self-developed Community Participation questionnaire, the SoC scale, and the Neuroticism subscale were used to assess CP, SoC, and neuroticism, respectively. The four indicators of SWB were assessed by the Satisfaction with Life Scale, Positive Affect and Negative Affect Schedule, and the Center for Epidemiological Studies-Depression scale. The results revealed that CP was associated with three indicators of SWB including life satisfaction, positive affect, and depressive symptoms, SoC mediated the above associations, and neuroticism negatively moderated the association between CP and SoC, after controlling for age, sex, education, spouse status, monthly income, and physical health. CP enhances older adults’ SWB through increasing their SoC. When compared to those older adults with a higher score of neuroticism, the enhancing effect of CP on SoC is stronger for those with lower scores of neuroticism.

## 1. Introduction

According to the convoy model, individuals continuously change their identities and roles, create new needs and expectations, and replace convoy memberships through their life-span and life-course [1,2]. Old age often means the shrinking of social relationship networks. For older adults, social participation is a necessary way to maintain social relations and size of convoy. With extended life expectancy and free post-retirement life, they have more opportunities to seek social support and social relations, and give their remaining life new meaning by participating in social community activities. The participation of older persons is an essential element and “golden standard” for active aging [3,4]. According to the seventh national census of China, the number of people aged 65 and above has accumulated to 191 million [5]. With the increasing number of the old adults’ population in China, how to improve the happiness of their later years has become the focus of the state and the government. In China, many old people remain active by participating in community affairs which benefit both themselves and residential community [6,7]. As the core content of active aging, community participation may play an important role in the subjective well-being of old adults.

According to the aging in place theory, older adults want to age in place, live with family and friends, and remain independent and autonomous; they also expect to have more opportunities to participate in social community activities which can help them form strong senses of security and belongingness of community [8,9]. The active community participation of older people is an important part of the realization of aging-in-place. Community participation (CP) refers to the fact that community members actively engage in and involve in those activities that affect their lives [10]. A recent study revealed that the social participation based on the community context of Chinese retirees included five aspects, which basically covered the main aspects of local community activities, met the needs of the physical condition and cognitive level of the older adults, and reflected the active degree of older adults participating in various community organizations or groups as members [11].

Despite the robust link between CP and subjective well-being (SWB) being well-established by a growing literature, understanding the basic mechanisms requires further efforts of researchers in different fields. Several studies emphasized the role of perceived support in the link between community engagement with well-being [12,13,14]. Some studies thought that meaning and purpose in life mediated the effect of community engagement (such as volunteering) on well-being [15,16]. According to previous studies, CP was closely associated with the feeling and attitude of residential community, the so called sense of community (SoC) [17]. The frequency and degree of participating into community affairs may reflect one’s belongingness to residential community, in turn also enhancing one’s belongingness to residential community [10]. SoC as a psychosocial factor may mediate the robust relationship between CP and SWB. A meta-analysis reviewed strength, stability, and variation of the relationship between CP and SoC, and showed that the association was generally positive in the adult population [10].

Although CP has been shown to be positively associated with SoC, this does not mean that all individuals can equally benefit from this process and contribute to growth of one’s SoC. Inferring from a person–environment interaction perspective [18], individual features can affect the effect of CP, such as age, education, economic status, length of residence, and so on. Comparatively speaking, few studies refer to the role of personality. Some researchers found that higher level of emotional stability was associated with higher level of SoC [19]. Thus, researchers need to learn whether the facilitating effect of CP on SoC of older adults is affected by neuroticism, which is typically characterized by emotional instability.

### 1.1. Community Participation and Sense of Community

For decades, SoC has become one of the most commonly used concepts and the focus of empirical research in field of community psychology. SoC is defined as “a perception of similarity to others, an acknowledged interdependence with others, a willingness to maintain this interdependence by giving to or doing for others what one expects from them, and the feeling that one is part of a larger dependable and stable structure” [20]. Some researchers think that SoC includes four components: membership, influence, integration and fulfillment of needs, and shared emotional connection [21]. SoC is often relevant to collective organizations or groups which individuals as members belong to or take part in; it involves a feeling of belongingness, cohesion, and connection as members living in shared residential environments [22,23].

Both CP and SoC are closely relevant to quality of life and mental health of community-dwelling residents, including older residents. A meta-analysis indicated a moderate association between CP and SoC; this correlation was quite stable across all studies included in the meta-analysis [10]. On the one hand, SoC is a catalyst or precondition of participating in community, such as civic engagement, volunteering behavior, and recreational activities [10,24]. On the other hand, CP influences the formation and growth of SoC [25,26,27]. For most of community-dwelling old residents, engaging in community affairs and joining community organizations (typically such as volunteering behaviors, public affairs of neighborhoods, or collective entertainment), can foster their needs of belonging, collective efficacy, solidarity, and cohesion, which represent main aspects of SoC [25,28].

### 1.2. Sense of Community and Subjective Well-Being (SWB)

Researchers have revealed the positive significance of SoC in influencing one’s mental health and SWB [29,30,31,32]. Some concepts similar to SoC or subcomponents of SoC, such as neighborhood collective efficacy or neighborhood cohesion, are associated with higher psychological well-being and lower depressive symptoms among older adults [33,34]. Using a longitudinally and nationally representative sample of adults aged >50, the study concluded that higher baseline neighborhood cohesion was associated with psychological well-being (e.g., higher life satisfaction and lower hopelessness), but not associated with most physical health indicators [34].

### 1.3. Neuroticism

Neuroticism is one of more foundational traits and reflects some kind of universal and cross-cultural personality tendency. According to Eysenck’s personality theory, introversion–extraversion and emotional stability–instability (or neuroticism) are two basic personality dimensions. Neuroticism reflects one’s emotional instability and is relevant to how people create or maintain available social networks [35]. The study revealed that nearly all dimensions of the Big Five were positively associated with SoC among adolescents and young adults, while neuroticism was negatively associated with SoC [19]. People with high scores of neuroticism seem more sensitive to interpersonal stressors and aspects of social interaction, and more likely to negatively appraise their social partners and context in the process of social participation [36,37]. That is, neuroticism may adversely influence one’s SoC or feeling for residential neighborhood and the association between CP and SoC. The study found that neuroticism predicted negatively lower levels of competence and enjoyment when participating in daily activities [38]. Using a follow-up survey, some researchers indicated that neuroticism was related directly to mental health and indirectly to life satisfaction through the mediating role of appraisals [39]. Although neuroticism is often as a moderator variable in etiology [40], much is yet unknown regarding whether the level of neuroticism affects the association between CP and SoC. Since relevant empirical studies on above moderating effect are scarce, the direction of moderating role is not explicitly specified in the study.

### 1.4. Theoretical Basis

The association between community participation and sense of community may be vary with individual features. Inferring from a perspective of person–environment interaction, a few intriguing studies have revealed the interaction effect of individual characteristics and community characteristics on SoC [18,41,42,43]. Some researchers pointed out that personal resources and individual’s involvement in community activity jointly influence one’s sense of community [42]. As mentioned above, personality traits as the fundamentally personal resources accompanying community participation as an environment resource were relevant to one’s sense of community; neuroticism may be the most likely to adversely affect one’s sense of community [19,44]. That is to say, during participating in community activities, individuals with different levels of neuroticism maybe have different feelings and attitudes on community.

According to the Diathesis–Stress model, the associations between environmental factors and psychological outcomes can be moderated by personal traits [45]. Personality as a typical kind of personal traits can affect how individuals perceive and interact with environmental stressors [46]. Residential community is the most important environmental context for older adults. When compared to other age groups, older adults often spend more time staying in their residential neighborhood. Participating in social organization and group can maintain and enhance sense of community or SWB of older adults [32,47,48]. Neuroticism is one of the most important personality traits in coping with environment stressors [49], and highly neurotic individuals are more vulnerable to be moody and influenced by life events [50,51]. Based on the above, the interaction between neuroticism and environmental factors (community) may affect individuals’ sense of community.

### 1.5. Present Study

Most existing studies have confirmed the close relationship between CP and SWB. However, the reason why CP affects the subjective well-being of old adults remains to be explored, in particular, the psychological mechanisms of the association between CP with Chinese characteristics and SWB of old people. It is worth noting that the old adults with different characteristics may achieve different degrees of SWB by participating in community activities. Thus, it is necessary to examine interaction effect between environmental factors and personality traits for the old people in the context of community.

The current study had three aims. First, we hoped to explore whether CP is associated with SWB (including life satisfaction, positive affect, negative affect, and depressive symptoms); second, whether SoC mediates above associations; third, whether neuroticism moderates the association between CP and SoC among old people. The hypotheses were as follows:

**Hypothesis** **1** (**H1**)**.** *CP is associated with SWB indicated by life satisfaction, positive affect, negative affect, and depressive symptoms*.

**Hypothesis** **2** (**H2**)**.** *SoC mediates the association between CP and SWB*.

**Hypothesis** **3** (**H3**)**.** *Neuroticism moderates the association between CP and SoC*.

## 2. Materials and Methods

### 2.1. Participants and Procedure

The sample was drawn from a national project on the organizational participation and quality of life of older adults. The ethics committees of the corresponding authors’ affiliation approved this project. Participants were provided online informed consent before the investigation was formally conducted. Community-dwelling residents aged 65 years and above were selected as participants from urban communities in five cities in China. The investigators adopted the convenience sampling to recruit participants by posting recruitment posters in the community and sending recruitment information in community Wechat groups. The investigators were trained to further ensure that they could understand the purpose of the survey and response participants’ questions. Participants answered questions on Questionstar—an online survey platform in China—due to prevalence of COVID-19 during the investigation process. After the questionnaires were collected, the investigators deleted some invalid questionnaires by screening whether the answers were the same or in order. The current project has completed two wave surveys with an interval of six months (the first wave survey was conducted in June 2020 and the second wave was conducted in December 2020). A total of 1576 valid questionnaires were collected in the first wave survey and 986 valid questionnaires were collected in the second wave survey. Finally, a total of 465 participants effectively completed both wave surveys. In the valid sample, there were 149 men and 316 women (see Table 1). Demographic and socioeconomic characteristics showed in Table 1 were from the first wave survey.

### 2.2. Instruments

#### 2.2.1. Community Participation Questionnaire

Community participation questionnaire referred to the community participation survey, with five items published in a recent study which included community residents’ organizations or neighborhood committees, volunteer service associations, wedding and funeral councils or similar organizations of residential communities, organizations based on interests, hobbies, and training, or community charitable organizations [11]. In our study, the fourth type of organization in the community participation survey (“organizations based on interests, hobbies, and training”) was subdivided into organizations based on indoor activities and outdoor activities according to activity venue. Hence, a community participation questionnaire with six items was applied in our study. Specifically, volunteer organizations are common community organizations in both Chinese and Western culture, while wedding and funeral councils are unique as a common form of non-governmental mutual aid organization in China.

Participants were asked to answer the level of participation in community organizations using a five-point scale (1 = never, 2 = occasionally, 3 = moderately, 4 = often, 5 = always). The reliability and validity of questionnaires were well verified. First, Cronbach’s alpha coefficient for all six items was 0.72, and coefficients of all pairwise correlations were positive and significant. Second, all participants were randomly divided into two equally sized sub-samples. Sub-sample 1 (n = 233) was used to conduct an exploratory factor analysis (EFA), and sub-sample 2 (n = 232) was used to conduct a confirmatory factor analysis (CFA). EFA showed that only one eigenvalue was above 1.0 (2.74); this single factor accounted for 45.72% of the total variance, and the factor loadings of all items were over 0.40. CFA indicated that the single-factor structure had an acceptable goodness of fit (χ^2^ = 13.52 (*p <* 0.001), χ^2^/df(8) = 1.69, CFI = 0.98, TLI = 0.96, RMSEA = 0.06 (90% CI: 0.00–0.10), SRMR = 0.03). As a result, the summated mean scores represented the overall level of CP of older adults in China.

#### 2.2.2. Sense of Community Scale

The SoC scale was drawn from the Community Social Capital survey, based on urban community residents of China, and its reliability and validity were satisfactory [52]. The SoC scale with 10 items captured the core meaning of sense of community [21] (e.g., “my neighborhood lets me have a feeling of home”). Items were rated on a 5-point Likert-type scale (1 = strongly disagree, 2 = disagree, 3 = moderate, 4 = agree, 5 = strongly agree). The aggregate mean score was used as the indicator of SoC. In the current study, Cronbach’s alpha coefficient for the scale was 0.92.

#### 2.2.3. Neuroticism

Neuroticism subscale came from the Midlife Development Inventory (MIDI) Personality Scales [53]. The neuroticism subscale had four items (“moody, worrying, nervous, calm (reverse scored)”), using a 5-point scale (1 = strongly disagree, 2 = disagree, 3 = moderate, 4 = agree, 5 = strongly agree). In the current study, Cronbach’s alpha coefficient for the neuroticism subscale was 0.75.

#### 2.2.4. Subjective Well Being

SWB is a comprehensive psychological index to measure individuals’ life quality and generally includes three aspects: life satisfaction, positive affect, and negative affect [54]. Since negative affect is characteristically low in older adults, depressive symptoms are used as a supplemental measure of negative affect, which makes the assessment of SWB more balanced. Hence, SWB in our study was measured by life satisfaction, positive affect, negative affect, and depressive symptoms [55,56].

#### 2.2.5. The Satisfaction with Life Scale (SWLS)

SWLS was used to assess one’s general satisfaction with life and reflects cognitive aspects of SWB [54]. The Chinese version of the SWLS demonstrated good reliability, validity, and invariance across the sexes [57]. In the current study, items were rated on a 5-point Likert-type scale (1 = strongly disagree, 2 = disagree, 3 = moderate, 4 = agree, 5 = strongly agree) (e.g., “In most ways my life is close to my ideal”), and Cronbach’s alpha coefficient for the scale was 0.85.

#### 2.2.6. Positive Affect and Negative Affect Schedule (PANAS)

The PANAS was often used to assess one’s general emotional respects of SWB and comprised positive and negative affect during the past month [58]. The Chinese version has shown good psychometric characteristics [59]. In the current study, a short version of the PANAS with 12 items (1 = not at all, 2 = slightly, 3 = moderately, 4 = very, 5 = extremely) was used, and each subscale consisted of 6 items [60]. In the current study, the Cronbach’s alpha coefficient was 0.86 for the PA and 0.85 for the NA, respectively.

#### 2.2.7. The Center for Epidemiological Studies-Depression (CES-D) Scale

CESD scale with 10 items was used to assess one’s depressive symptoms during the last month [61]. The responses were on a four-scale metric (0 = less than one day, 1 = one-two days, 2 = three-four days, and 3 = five-seven days). Higher average scores mean greater depressive symptoms. Chinese version has been conformed to be suitable to assess depressive symptoms of older adults [62]. In the current study, Cronbach’s alpha coefficient was 0.83.

#### 2.2.8. Covariates

Demographic and socio–economic factors as covariates included age, sex, education, spouse status, monthly income, and physical health. Overall physical health was assessed by a single-item: “Compared to people your own age, please rate your physical health on the whole” (1 = poor, 2 = fair, 3 = good, and 4 = excellent). Participants reported individual monthly income as the indicator of economic status with a 5-grade income list (from “less than 2000 CNY” to “more than 8000 CNY”). The grade scores of physical health and monthly income were treated as continuous variables in the analysis (see Table 1).

### 2.3. Statistical Analysis

SPSS 24.0 version (IBM, Armonk, NY, US) program was used to examine the descriptive statistics and correlation analyses. Mplus 8.0 [63] was employed to test the structural equation model, and the maximum likelihood estimation was adopted to estimate the mediation model and moderated mediation model. The CP, as the independent variable, and covariates were investigated in the first wave; SoC as the mediating variable, neuroticism as the moderating variable, and four indicators of subjective well-being simultaneously as dependent variables were measured in the second wave. It is worth noting that four indicators of subjective well-being were simultaneously used as dependent variables in the model, and dependent variables and the mediating variable were adjusted with the covariates. Several common indicators of model fitness were used to evaluate goodness-of-fit of the structural model [64]. These indices included the Chi square statistic value (χ^2^) and freedom degree, CFI, TLI, RMSEA, and SRMR.

## 3. Results

### 3.1. Descriptive Findings

On the whole, participants reported a moderate general level of CP (*M* = 2.36, *SD* = 0.64). About 64.9% of older adults reported a relatively high level of participation in at least one type of community organization (ratio of selecting “4 = often” or “5 = always”). Participants had higher sense of community, life satisfaction and positive affect, lower depressive symptoms and negative affect, and moderate neuroticism when compared to the middle values (see Table 2).

### 3.2. Correlation Analyses

There were significant pairwise correlations between CP, SoC, neuroticism personality, and SWB. First, there were relatively low but significant correlations between CP and three indicators of SWB, including life satisfaction, positive affect, and depressive symptoms. However, CP was not significantly associated with negative affect. Hypothesis 1 was partially confirmed. Second, CP was significantly associated with SoC. Third, SoC was significantly associated with all indicators of SWB. In addition, some demographic and socio-economic factors were related to one or several core variables. Thus, it was necessary to control the potential confounding roles of these factors during data analyses (see Table 2).

### 3.3. Structure Equation Model

The goodness of fit of the mediation model was saturated. The results indicated that SoC mediated the relationship between CP and SWB; Hypothesis 2 was confirmed. That is to say, indirect effects of CP on all indicators of SWB through SoC were significant. Direct effects of all four indicators of SWB were not significant, which meant that SoC completely mediated the relationships between CP and SWB (See Table 3). All results were standardized values.

**Figure 1:**
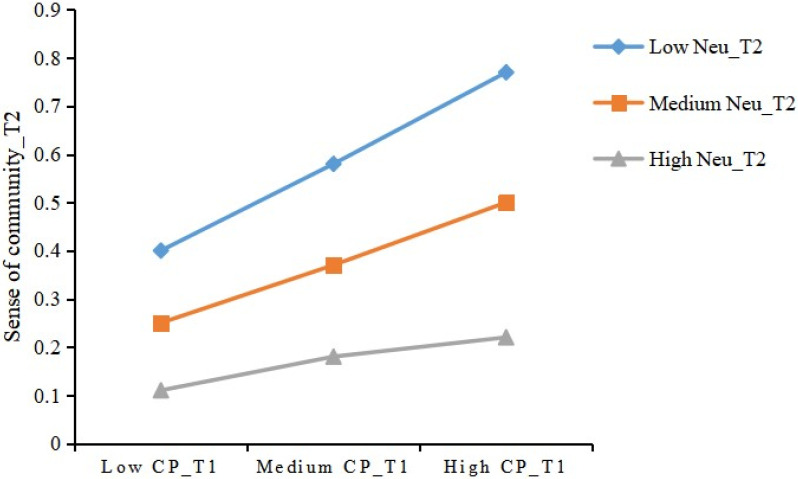
Community participation and sense of community: Moderating role of neuroticism. *Note.* CP and Neu represent community participation and neuroticism.

**Figure 2:**
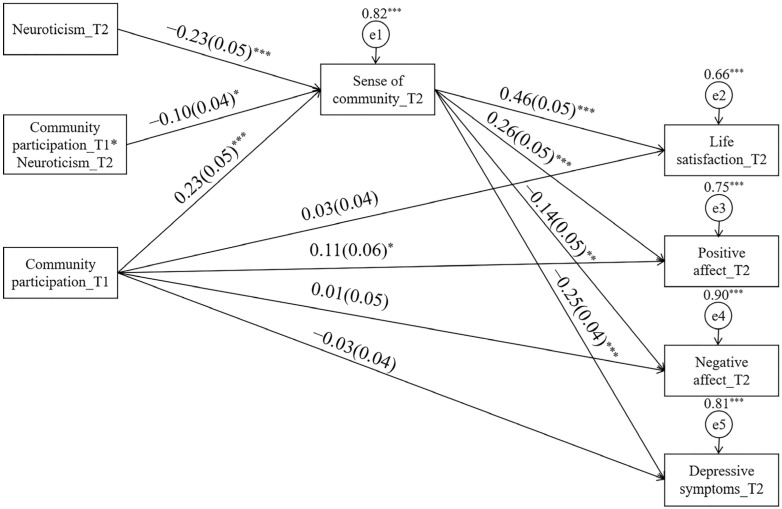
Community participation and subjective well-being: A moderated mediation model. *Note*. * *p* < 0.05; ** *p* < 0.01; *** *p* < 0.001.

We conducted the moderated mediation model analysis after a mere mediation model. The goodness of fit of the moderated mediation model was as follows: χ^2^ = 70.97 (*p* < 0.001), χ^2^/df(22) = 3.23, CFI = 0.97, TLI = 0.91, RMSEA = 0.07 (90% CI: 0.06–0.09), SRMR = 0.06. The goodness of fit was not excellent but basically acceptable [65]. The results indicated that interaction effect of CP and neuroticism on SoC was significant and negative. Hypothesis 3 was confirmed. Conditional effect at three levels of neuroticism further supplemented this finding. Effect of CP on SoC was significant only when neuroticism was in the low to middle level. When compared to those older adults with a higher score of neuroticism, the enhancing effect of CP on SoC was stronger for those with lower score of neuroticism (see Table 4 and Figure 1 and Figure 2).

## 4. Discussion

### 4.1. Community Participation, Sense of Community and Neuroticism

The findings provide a new perspective for exploring paths and conditions whereby CP affects SWB. Meanwhile, policy-makers and managers can take advantage of these insightful findings to advance active aging in basic-level community.

First, Hypothesis 1 was partly confirmed. The older adults having a higher frequency of CP often reported higher life satisfaction and positive affect, and lower depressive symptoms. The benefits of CP were confirmed again, which were consistent with previous main findings. As the Declaration of Madrid on aging emphasizes, participation, accompanying health and security, is one of core standards of active and successful aging [4]. The results further support the aging-in-place theory. To some extent, the involvement level of older people in CP represents the realization degree of aging-in-place and the frequency of interaction among community members. Active community participation is beneficial to develop a sense of belonging, community attachment, and a sense of environmental control, which in turn contribute to positive mental health and well-being of older adults [8,9]. In addition, CP may contribute to one’s SWB through its two attributes: functionality and sociality [66]. Functionality gives seniors the freedom to do what they are interested in. Sociality offers them more opportunities to build new social ties and expand size or diversity of social network. With the two attributes, CP enhances one’s SWB.

Second, Hypothesis 2 was confirmed. SoC, to a large extent, accounts for the effects of CP on nearly all four indicators of SWB. Some relevant theories and empirical studies give some inspirations from different perspectives. Firstly, psychological empowerment theory believes that CP empowers individuals with sense of competence or self-control, and enables them to independently cope with affairs of their residential community [67]. Extant studies generally showed that CP, SoC, and the sense of empowerment are closely related to and influence one another; and SoC is an important empowering organizational characteristic [68]. Secondly, in review of social capital theory, CP can enhance one’s social capital and network, which has a closely internal link to SoC [20,69]. CP can expand the size and diversity of the social networks of old people, which results in a more diverse support, and a stronger sense of belonging to residential community [70]. For old people, active CP partly offsets the negative impact of losing job roles and shrinking social networks by joining a new organization or getting acquainted with new residents [71]. Inferring from self-determination theory, CP can simultaneously satisfy three basic psychological needs of the older population: autonomy, competence, and relatedness, and lead to the formation of SoC and the increase of SWB [72,73]. CP meets older adults’ relatedness needs by expanding one’s social communication and strengthening one’s neighborhood bond. Community organization provides older adults a platform to resume their self-autonomy and sense of competence as members of community. The older adults feel that they make an impact on society by resolving neighborhood problems and contributing their own remaining energy. For example, “neighborhood committee aunt” has become an informal title, which specifically refers to those older women who assist in community governance and service. All these theoretical explanations are complementary and interrelated to each other and need further testing.

Third, Hypothesis 3 was confirmed. Neuroticism negatively moderated the effect of CP on SoC. We also found that neuroticism is negatively associated with SoC. Emotional instability as adverse side of neuroticism is related to the sense of belonging, attachment to community, and reciprocal trust among residents [38,74]. Relevant studies find that neuroticism predicts negatively lower levels of sense of competence and enjoyment during social participation, and that neuroticism is related directly to mental health and indirectly to life satisfaction through the mediating role of appraisals [38,39]. The level of neuroticism in older adults could affect their performance and feelings in social activities. When compared to those older adults with higher score of neuroticism, the enhancing effect of CP on SoC is stronger for those with lower scores of neuroticism. The conditional effect analysis indicated that low and medium levels, but not high levels, of neuroticism personality can contribute to SoC of older adults. From the perspective of person–environment interaction, the individuals’ SoC formed in community environment is affected by their neuroticism level. Additionally, the higher the level of neuroticism, the more adversely affected the SoC [19,42]. From the perspective of the Diathesis–Stress model, neuroticism moderates the relationship between environmental factors and individual psychology outcomes [45,46]. Community environment, as an important scene of the life and activity of older adults, and individual neuroticism levels jointly determine the degree of SoC of older residents.

### 4.2. Theoretical and Practical Significance

It should be acknowledged that the current study has unique theoretical and practical contributions. First, despite a mass of evidence having confirmed the positive benefits of community participation on enhancing SWB, the relevant mechanisms based on residential community remain insufficient in the Chinese context. These findings offer further evidence for the aging-in-place theory and provide the direction for construction and implementation of age-friendly communities, which contribute to active aging. Second, this study explored the determinants of SoC from individual characteristics and social context. The findings of the current study further supplement person–environment interaction thought, based on specific personality and community organization and the Diathesis–Stress model [43,45]. The neuroticism trait is closely related to environmental factors, and their interaction can further affect individuals’ psychological feelings or behavioral results. Finally, from a practical point of view, policy-makers and the managers of basic-level government and community should take into account the significance of participation and its psychological significance for the older population when laying down measures and policies to improve older adults’ well-being; and create conditions to advance and encourage their social participation based on real conditions of residential community and individual traits of older adults.

### 4.3. Limitations

Several limitations in the design and methods should be pointed out. First, we adopted a two-wave longitudinal design with six months apart to examine the association between CP and SWB and relevant mediating and moderating mechanisms. Some scholars have proposed that conducting a complete mediating analysis needs at least three occasions of measurement, and adopted a cross-lagged analysis [75,76]. Therefore, the possibility of reverse causality between variables cannot be ruled out based on an observational analysis. Additionally, whether the interval time of this study leads to meaningful changes in individuals’ behavior is also a limitation of this study. Second, the sample in the current study mainly came from urban areas, who might not be representative of the overall status of the older population in China. Furthermore, given prevalence of COVID-19, researchers could only adopt the mode of online survey to collect data, which limited the generalization of findings. That is to say, the results of the study may not generalize to the scenario outside the pandemic. If possible, future research will adopt a more representative sample and a more scientific sampling technique. Third, the beneficial role of CP is repeatedly confirmed; however, this does not mean that the negative side of CP should be neglected. Some findings have indicated that social participation has duality and provides a mix of positive and negative impacts [77]. For example, involvement in community affairs not only enhances social integration but also triggers some interpersonal conflicts. This reminds future researchers that this duality is of crucial importance for a comprehensive understanding of how CP affects SWB.

## 5. Conclusions

This study is a meaningful exploration to understand the significance of CP and the relevant mechanisms in maintaining and enhancing SWB among older adults. First, the findings supplement further evidence about benefits of CP on mental health and quality of life among older adults. Second, SoC as a psych-socio factor mediates the link between CP and SWB. Third, there were individual differences in the benefits of CP on SoC. Limitations and questions surrounding current study would provide direct guidance for future studies in field of community and aging psychology. These findings and thoughts will also provide some insights for community management and services on successful and healthy aging.

## Figures and Tables

**Table 1:** Demographic and socioeconomic characteristics.

Variable	*n*	Valid %
Gender		
Male	149	32.04
Female	316	67.96
Age (years)		
65~69	290	62.37
70~74	134	28.82
75~79	32	6.88
80 and over	9	1.94
Educational level		
Junior high school and lower	58	12.47
Senior high school	112	24.09
Junior college	170	36.56
Bachelor degree or above	125	26.88
Spouse status		
Without a spouse	78	16.77
With a spouse	387	83.23
Monthly income (CNY)		
Less than 2000	28	6.02
2000~4000	158	33.98
4000~6000	99	21.29
6000~8000	58	12.47
More than 8000	122	26.24
Physical condition		
Poor	10	2.15
Fair	211	45.38
Good	207	44.52
Excellent	37	7.96

Note. CNY represents Chinese Yuan.

**Table 2:** Inter-correlations between main variables.

	*M*(*SD*)	1	2	3	4	5	6	7	8	9	10	11	12
1 Age		1											
2 Sex		0.01	1										
3 Educational level		0.04	−0.26 ***	1									
4 Marital status		0.13 **	0.11 *	−0.01	1								
5 Monthly income		0.13 **	−0.14 **	0.37 ***	0.04	1							
6 Physical condition		−0.02	−0.02	0.02	0.02	0.05	1						
7 Community participation_T1	20.36(0.64)	−0.02	0.05	−0.02	−0.05	0.01	0.09	1					
8 Neuroticism_T2	20.54(0.64)	−0.05	−0.01	−0.10 *	−0.03	−0.15 **	−0.30 ***	−0.14 **	1				
9 Sense of community_T2	30.69(0.63)	0.01	0.13 **	−0.04	−0.06	0.04	0.26 ***	0.28 ***	−0.30 ***	1			
10 Life satisfaction_T2	30.76(0.69)	0.06	0.13 **	0.07	−0.09 *	0.16 **	0.28 ***	0.18 ***	−0.41 ***	0.57 ***	1		
11 Positive affect_T2	30.46(0.65)	0.02	0.09 *	0.13 **	−0.05	0.06	0.42 ***	0.20 ***	−0.53 ***	0.45 ***	0.54 ***	1	
12 Negative affect_T2	10.54(0.50)	−0.02	0.01	0.03	0.04	−0.01	−0.33 ***	−0.05	0.57 ***	−0.31 ***	−0.44 ***	−0.45 ***	1
13 Depressive symptoms_T2	0.77(0.53)	−0.02	−0.05	−0.06	0.05	−0.05	−0.39 ***	−0.12 *	0.59 ***	−0.43 ***	−0.51 ***	−0.65 ***	0.75 ***

Note. * *p* < 0.05; ** *p* < 0.01; *** *p* < 0.001.

**Table 3:** Total effect, direct effect, and indirect effect.

	β	*Se*	*t*	*p*	Effect Size	LLCL	ULCL
From CP_T1 to LS_T2							
Total effect	0.14	0.04	3.33	0.001		0.05	0.22
Direct effect	0.02	0.04	0.40	0.687	13.33%	−0.06	0.09
Indirect effect	0.13	0.03	4.80	0.000	86.67%	0.08	0.18
From CP_T1 to PA_T2							
Total effect	0.16	0.05	3.02	0.003		0.06	0.27
Direct effect	0.08	0.05	1.49	0.135	47.06%	−0.02	0.18
Indirect effect	0.09	0.02	4.40	0.000	52.94%	0.05	0.13
From CP_T1 to NA_T2							
Total effect	−0.01	0.04	−0.33	0.744		−0.10	0.07
Direct effect	0.05	0.04	1.16	0.248	45.45%	−0.03	0.13
Indirect effect	−0.06	0.02	−3.81	0.000	54.55%	−0.10	−0.03
From CP_T1 to DEP_T2							
Total effect	−0.08	0.04	−1.80	0.073		−0.17	0.03
Direct effect	0.01	0.04	0.21	0.834	10.00%	−0.08	0.09
Indirect effect	−0.09	0.02	−4.73	0.000	90.00%	−0.13	−0.06

Note. CP, LS, PA, NA, and DEP represent community participation, life satisfaction, positive affect, negative affect, and depressive symptoms, respectively.

**Table 4:** Conditional effects of CP on SoC at three levels of neuroticism.

Three Levels of Neuroticism	β	*Se*	*t*	*p*	LLCI	ULCI
M − 1SD	0.33	0.06	5.65	0.000	0.21	0.43
M	0.22	0.05	4.82	0.008	0.13	0.31
M + 1SD	0.12	0.07	1.78	0.074	−0.02	0.24

Note. CP and SoC represent community participation and sense of community.

## Data Availability

The datasets used and/or analyzed during the current study are available from the corresponding author on reasonable request.
